# Clinical features of hypermagnesemia in patients with functional constipation taking daily magnesium oxide

**DOI:** 10.3164/jcbn.18-117

**Published:** 2019-07-01

**Authors:** Hideki Mori, Hidekazu Suzuki, Yuichiro Hirai, Anna Okuzawa, Atsuto Kayashima, Yoko Kubosawa, Satoshi Kinoshita, Ai Fujimoto, Yoshihiro Nakazato, Toshihiro Nishizawa, Masahiro Kikuchi

**Affiliations:** 1 Department of Gastroenterology, National Hospital Organization Tokyo Medical Center, 2-5-1 Higashigaoka, Meguro-ku, Tokyo 152-8902, Japan; 2 Department of Gastroenterology, Tokai University School of Medicine, 143 Shimokasuya, Isehara, Kanagawa 259-1193, Japan; 3 Digestive Disease Center, International University of Health and Welfare, Mita Hospital, 1-4-3 Mita, Minato-ku, Tokyo 108-8329, Japan

**Keywords:** magnesium oxide, hypermagnesemia, chronic kidney disease, age

## Abstract

Although magnesium oxide is widely used as a laxative, alterations in serum magnesium concentrations among patients taking daily magnesium oxide have not been clarified. The present retrospective, cross-sectional study investigated the risk factors for hypermagnesemia in patients taking daily oral magnesium oxide. Of 2,176 patients administered daily magnesium oxide, 193 (8.9%) underwent assays of serum magnesium concentrations and were evaluated. High serum magnesium concentration and hypermagnesemia were defined as serum magnesium concentrations ≥2.5 mg/dl and ≥3.0 mg/dl, respectively. Of the 193 patients taking daily magnesium oxide, 32 (16.6%) had high serum magnesium concentration and 10 (5.2%) had hypermagnesemia. Factors associated with hypermagnesemia included chronic kidney disease (CKD) grade 4 (*p* = 0.014) and magnesium oxide dosage (*p* = 0.009). Factors associated with high serum magnesium concentration included magnesium oxide dosage >1,000 mg/day (*p* = 0.004), CKD grades 4 (*p* = 0.000) and concomitant use of stimulant laxatives (*p* = 0.035). Age, however, was not associated with hypermagnesemia or high serum magnesium concentration. In conclusion, renal function and magnesium oxide dosage, but not age, were associated with hypermagnesemia and high serum magnesium concentration in patients with functional constipation taking daily magnesium oxide.

## Introduction

The ability of magnesium oxide to induce bowel movements by drawing water into the large intestine^(1)^ has led to its widespread use as a laxative to treat constipation.^(2)^ Its poor bioavailability makes magnesium oxide relatively safe, with an estimated 10 million patients in Japan treated with this agent for constipation.^(3)^ Prolonged treatment with magnesium oxide, however, may induce hypermagnesemia.^(4–9)^ Serum magnesium concentrations >5.0 mg/dl have been associated with nausea, headache, light-headedness, and cutaneous flushing, whereas levels above 12 mg/dl have been associated with respiratory failure, complete heart blockage, and cardiac arrest.^(10)^ Recently, the Japanese Ministry of Health, Labour and Welfare recommended that serum magnesium concentrations be measured periodically in geriatric patients and in patients administered magnesium oxide for prolonged periods of time (http://www.pmda.go.jp/files/000219708.pdf). However, alterations in serum magnesium concentrations among patients with functional constipation taking daily magnesium oxide have not been clarified. The present study investigated the risk factors for high magnesium concentration and hypermagnesemia in patients receiving oral magnesium oxide.

## Subjects and Methods

### Study population

Data were obtained retrospectively from the medical records of patients administered magnesium oxide as a laxative for functional constipation at National Hospital Organization Tokyo Medical Center from September to December 2017. Doses of magnesium oxide, duration of magnesium oxide treatment, total dosage of magnesium oxide, serum concentrations of magnesium and creatinine, age, gender, and concomitant use of other laxatives were recorded. Patients were excluded if they had received magnesium sulfate to prevent preterm labor. Patients were divided into three groups. Patients in whom serum concentrations of creatinine or magnesium were not measured were defined as Group A; patients in whom serum concentrations of creatinine, but not magnesium, were measured were defined as Group B; and patients in whom blood tests were performed that included serum concentrations of creatinine and magnesium were defined as the Per Protocol Set.

This study protocol was approved by the Committee for Medical Ethics of National Hospital Organization Tokyo Medical Center (Approval ID No. R18-015; April 23, 2018) and was conducted in accordance with the tenets of the Declaration of Helsinki 1975 and its later amendment in 1983. Specific written informed consent was not obtained, as informed consent included an opt-out clause according to the Medical Ethics Committee. The study was observational, and its results have been reported in accordance with the Strengthening Reporting of Observational Studies in Epidemiology guidelines.^(11)^

### Study design

High serum magnesium concentration and hypermagnesemia were defined as serum magnesium concentrations ≥2.5 mg/dl and ≥3.0 mg/dl, respectively. Factors assessed for their association with high serum magnesium concentration and hypermagnesemia included renal function, daily magnesium oxide dose, duration of treatment with magnesium oxide, total dosage of magnesium oxide, age, gender, and concomitant use of other laxatives. Renal function was evaluated by measuring estimated glomerular filtration rate (eGFR) and chronic kidney disease (CKD) stage.^(12)^ CKD stage was divided into six categories, depending on eGFR, with stages G1, G2, G3a, G3b, G4, and G5 defined as eGFRs ≥90, 60–89, 45–59, 30–44, 15–29, and <15 ml/min/1.73 m^2^, respectively. Elderly patients were defined as those aged ≥75 years.^(13)^ Stimulant laxatives included anthraquinones (e.g., senna) and diphenylmethanes (e.g., bisacodyl, sodium picosulfate), and chloride channel activators included lubiprostone and linaclotide.

### Statistical analysis

All data were expressed as means ± SDs. Factors associated with high serum magnesium concentration and hypermagnesemia were evaluated by Fisher’s exact test, the Jonckheere-Terpstra trend test, and Spearman’s rank correlation test, as appropriate. Univariate logistic regression analysis was used to evaluate factors associated with hypermagnesemia and high serum magnesium concentration. Factors that showed a marginally significant association (*p*<0.1) in univariate analysis were subsequently evaluated by multivariate logistic regression analysis. All statistical analyses were performed using SPSS 25 for Windows (SPSS Inc., Chicago, IL).

## Results

### Patient characteristics

**Fig. 1:**
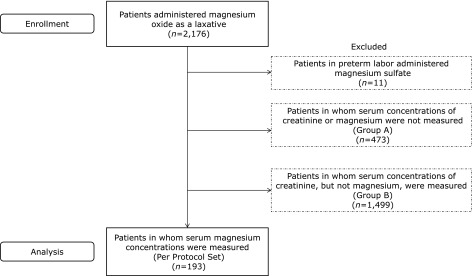
Flow diagram of study subjects.

A total of 2,176 patients were administered magnesium oxide as a laxative during the study period. Of these, 1983 were excluded, including 11 in preterm labor who were administered magnesium sulfate, 473 in whom serum concentrations of creatinine or magnesium were not measured (Group A), and 1,499 patients in whom serum concentrations of creatinine, but not serum magnesium, were measured (Group B). Finally, 193 patients were included in the Per Protocol Set and analyzed (Fig. 1). The mean ± SD age of patients in the Per Protocol Set was 73.2 ± 14.6 years, greater than those of patients in Groups A (65.0 ± 19.3 years, *p* = 0.000) and B (69.7 ± 16.0 years, *p* = 0.003). The percentage of males was significantly higher in the Per Protocol Set than in Group A (51.8% vs 36.6%, *p* = 0.000). Daily doses of magnesium oxide in the Per Protocol Set (1,040 ± 430 mg) did not differ significantly from those in Groups A (1,024 ± 453 mg, *p* = 0.667) and B (1,014 ± 509 mg, *p* = 0.498). However, duration of treatment with magnesium oxide (31.9 ± 50.4 months vs 49.1 ± 55.6 months, *p* = 0.000) and total dosage of magnesium oxide (1,019 ± 1,974 g vs 1,748 ± 2,621 g, *p* = 0.000) were significantly lower in the Per Protocol Set than in Group A. Mean eGFR was significantly lower in the Per Protocol Set than in Group B (63.3 ± 29.8 ml/min/1.73 m^2^ vs 69.9 ± 36.6 ml/min/1.73 m^2^, *p* = 0.010). Of the patients in the Per Protocol Set, 21 (10.9%) had an eGFR <30 ml/min/1.73 m^2^ (CKD stage 4 or 5), 47 (24.3%) had an eGFR <45 ml/min/1.73 m^2^ (CKD stage 3b to 5), and 96 (49.7%) had an eGFR <60 ml/min/1.73 m^2^ (CKD stage 3a to 5) (Table 1). The average serum magnesium concentration among patients in the Per Protocol Set was 2.21 mg/dl [95% confidence interval (CI), 2.15–2.67 mg/dl], with the highest magnesium level observed being 4.5 mg/dl. Ten patients (5.2%) had hypermagnesemia, defined as serum magnesium ≥3.0 mg/dl, and 32 (16.6%) had high serum magnesium concentration, defined as serum magnesium ≥2.5 mg/dl. None of these patients experienced symptoms of hypermagnesemia, such as nausea, headache, light-headedness, or cutaneous flushing.

### Baseline factors associated with hypermagnesemia/high serum magnesium concentration

The only factor significantly associated with hypermagnesemia among patients in the Per Protocol Set was CKD grade 4 (eGFR <30 ml/min/1.73 m^2^, *p* = 0.014), whereas factors marginally associated with hypermagnesemia were magnesium oxide dosage >1,000 mg/day (*p* = 0.053) and age >75 years (*p* = 0.098) (Table 2). Factors associated with high serum magnesium concentration included magnesium oxide dosage >1,000 mg/day (*p* = 0.004), CKD grade 4 (*p* = 0.000), and concomitant use of stimulant laxatives (*p* = 0.035), with age >75 years (*p* = 0.070) being marginally associated with high serum hypermagnesium concentration (*p* = 0.070) (Table 3). Multivariate logistic regression analysis showed that magnesium oxide dosage [*p* = 0.003; odds ratio (OR), 12.70; 95% CI, 2.43–66.48] and CKD grade 4 (*p* = 0.000; OR, 7.71; 95% CI, 2.46–24.19) were independently associated with high serum magnesium concentration (Table 3).

**Fig. 2:**
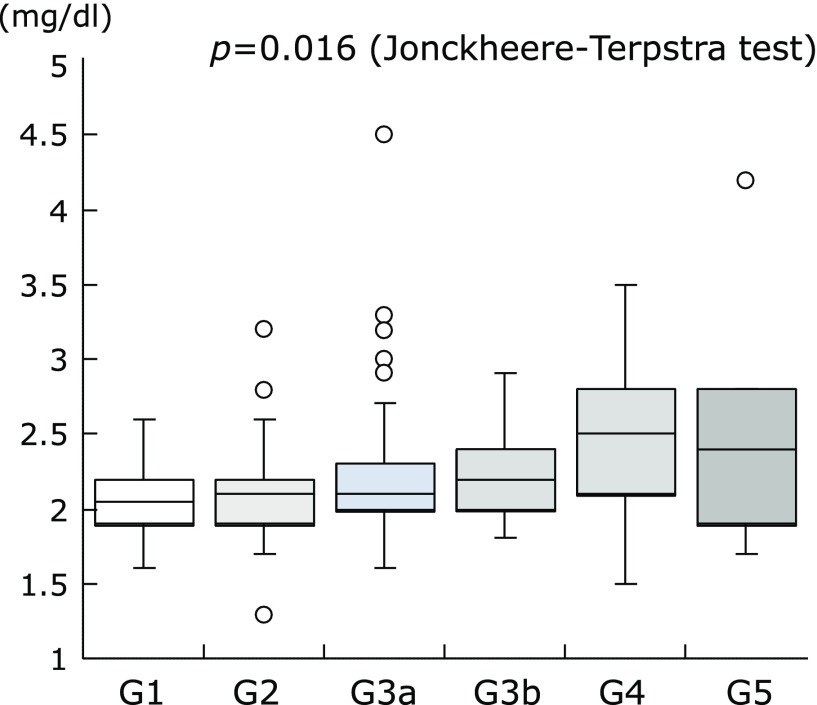
Correlation between serum magnesium concentration and CKD stage. A significant trend was observed between lower serum magnesium concentration and higher CKD stage (*p* = 0.016 by the Jonckheere-Terpstra test). CKD, chronic kidney disease.

**Fig. 3:**
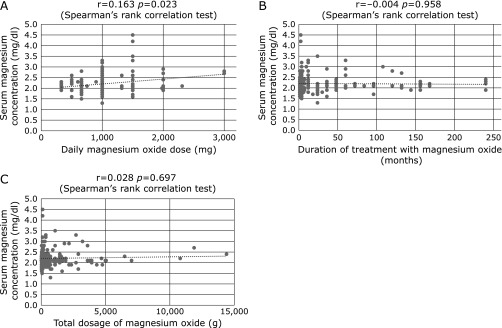
Correlations by Spearman’s rank correlation test between serum magnesium concentration and (A) magnesium oxide dosage, (B) duration of magnesium oxide treatment, and (C) total dosage of magnesium oxide. A significant linear association was observed between magnesium oxide dosage and serum magnesium concentration (r = 0.163, *p* = 0.023). However, serum magnesium concentration was not significantly correlated with duration of magnesium oxide treatment (r = –0.004, *p* = 0.958) or total dosage of magnesium oxide (r = 0.028, *p* = 0.697).

**Fig. 4:**
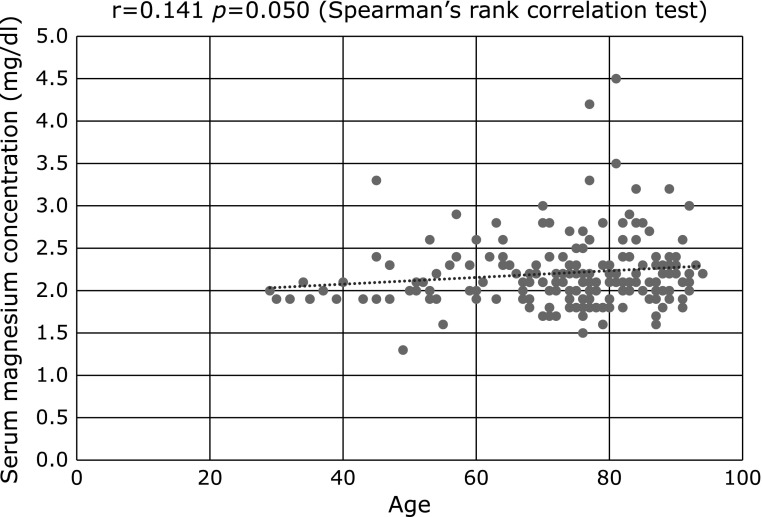
Correlation between serum magnesium concentration and age. A marginally significant linear association was observed between patient age and serum magnesium concentration (r = 0.141, *p* = 0.050 by Spearman’s rank correlation test).

The Jonckheere-Terpstra test showed a significant trend between lower serum magnesium concentration and greater CKD stage (*p* = 0.016, Fig. 2). Spearman’s rank correlation test showed a significant linear association between magnesium oxide dosage and serum magnesium concentration (r = 0.163, *p* = 0.023, Fig. 3A). By contrast, serum magnesium concentration was not associated with duration of treatment with magnesium oxide (r = −0.004, *p* = 0.958, Fig. 3B) or with total dosage of magnesium oxide (r = 0.028, *p* = 0.697, Fig. 3C). By contrast, serum magnesium concentration was marginally associated with age (r = 0.141, *p* = 0.050, Fig. 4). In elderly patients aged ≥75 years with CKD grades G1, G2, G3a, G3b, G4, and G5, the rates of hypermagnesemia were 0.0% (0/9), 3.4% (1/29), 10.8% (4/37), 0.0% (0/17), 20.0% (2/10), and 20.0% (1/5), respectively, and the rates of high serum magnesium concentration were 0.0% (0/9), 13.8% (4/29), 16.2% (6/37), 23.5% (4/17), 60.0% (6/10), and 40.0% (2/5), respectively. In younger patients (i.e., those aged <75 years) with CKD grades G1, G2, G3a, G3b, G4, and G5, the rates of hypermagnesemia were 0.0% (0/23), 0.0% (0/36), 8.3% (1/12), 0.0% (0/9), 16.7% (1/6) and 0% (0/0), respectively, and the rates of high serum magnesium concentration were 4.3% (1/23), 5.6% (2/36), 33.3% (4/12), 11.1% (1/9), 33.3% (2/6) and 0% (0/0), respectively. The rates of hypermagnesemia in patients with CKD grades G1 and G2 and the rates of high serum magnesium concentration in patients with CKD grade G1 were very low, even in elderly patients.

## Discussion

High CKD, greater age, and long-term use of magnesium oxide were shown to be risk factors for hypermagnesemia.^(3,5–10)^ Most of these studies, however, were case reports or studies of certain groups, including elderly patients, children, and patients undergoing hemodialysis. The results of the present retrospective cohort study showed that reduced renal function was the most important risk factor for hypermagnesemia and high serum magnesium concentration in patients taking magnesium oxide for functional constipation. These results also showed that high magnesium oxide dosage and concomitant use of stimulant laxatives may affect magnesium homeostasis, whereas age, gender, and long-term use of magnesium do not.

The kidneys play a central role in magnesium homeostasis through active reabsorption, which is influenced by the sodium load in the tubules and possibly by acid-base balance.^(14)^ The kidneys of individuals with a normal GFR filter approximately 2,000–2,400 mg of magnesium per day.^(14)^ In this study, the average serum magnesium concentration in patients with CKD grade was 2.1 mg/dl (range, 1.6–2.6 mg/dl), close to normal limits. By contrast, 38% of patients with CKD stages 4–5 had high serum magnesium concentrations, and 19% had hypermagnesemia. These results suggest the importance of monitoring serum magnesium levels in patients being treated with magnesium, especially in patients with chronic kidney failure.

The relationship between magnesium oxide dosage and serum magnesium concentration has not yet been clarified. Although case studies have reported that large dosages of oral magnesium were associated with hypermagnesemia,^(15,16)^ magnesium citrate dosage did not correlate with serum magnesium concentration.^(17)^ Our results showed that magnesium oxide dosage is an important and dose-dependent risk factor for high serum magnesium concentration and hypermagnesemia. Patients administered ≥1,000 mg/day magnesium oxide, especially those with impaired renal function, should therefore be monitored for possible hypermagnesemia. By contrast, we found that magnesium doses <1,000 mg/day were relatively safe.

Serum magnesium concentration was not significantly associated with the duration of magnesium oxide treatment or with the total dosage of magnesium oxide. Few studies have assessed the relationship between hypermagnesemia and duration of magnesium oxide treatment or total dosage of magnesium oxide, although a recent study showed that magnesium oxide administration for ≥36 days was a risk factor for hypermagnesemia.^(18)^ Patients in that study took magnesium oxide for <1 year, but longer-term treatment with magnesium was not evaluated. Additional prospective investigations may be required to assess the relationship between duration of magnesium oxide treatment and hypermagnesemia.

Our results also suggest that concomitant administration of stimulant laxatives in patients being treated with magnesium may be a risk factor for high serum magnesium concentration and hypermagnesemia. Serum magnesium concentration in constipated children with normal renal function was found to increase significantly after daily treatment with magnesium oxide.^(19)^ In some patients, severe hypermagnesemia is caused by a bowel motility disorder.^(20,21)^ Taken together, these findings suggest that serum magnesium concentration should be monitored regularly in patients with intestinal motility disorders who require stimulant laxatives.

In contrast to the above factors, age showed little correlation with serum magnesium concentration. Interestingly, the percentages of elderly patients without renal dysfunction who had hypermagnesemia and high serum magnesium concentration were very low and equal to those in younger patients. Advanced age has been reported to be a risk factor for hypermagnesemia in patients administered magnesium oxide.^(22–24)^ Some elderly individuals experience reduced renal function and a decrease in gastrointestinal motility.^(25,26)^ Chronic diseases in elderly individuals, such as long-standing diabetes mellitus, depression, hypothyroidism, and chronic renal failure, may prolong bowel transit time,^(27–30)^ suggesting the importance of determining medical history in elderly patients treated with magnesium.

Limitations of this study included its retrospective design and performance at a single institution. Many patients were excluded because their serum magnesium concentrations were not measured during the study period. Several possible reasons may explain the absence of blood tests in so many patients: First, younger individuals and those with normal renal function may not have been evaluated because they were judged to be at low risk of hypermagnesemia. Indeed, the mean age of the Per Protocol Set was higher than those of Groups A and B, and the mean eGFR of the Per Protocol Set was lower than that of Group B. Second, many clinicians may be unaware of the risk of hypermagnesemia in patients taking magnesium oxide. Accurate evaluation of patient backgrounds and the risks of hypermagnesemia requires distinguishing between chronic constipation and constipation predominant irritable bowel syndrome (IBS). However, it was difficult in this study to distinguish between chronic constipation in a narrow sense and constipation predominant IBS because the interview method has not been standardized for chronic constipation; that is, the interviewers did not always ask patients about abdominal pain/discomfort or lumpy/hard stools. Further prospective investigations will be necessary to confirm and extend these results.

In conclusion, this study showed that renal function and magnesium oxide dosage, but not age, were associated with hypermagnesemia in patients taking daily magnesium oxide for functional constipation.

## Figures and Tables

**Table 1:** Demographic and clinical characteristics of patients in this study

		Per Protocol Set	Group A	Group B	*p* value^a^	*p* value^b^
Total number, *n*		193	473	1,499		
Mean age [years (mean ± SD)]		73.2 ± 14.6	65.0 ± 19.3	69.7 ± 16.0	0.000**	0.003**
Age [older (≥75 years)/younger (<75 years)]		107/86	204/269	701/798	0.001**	0.026*
Gender (male/female)		100/93	173/300	667/832	0.000**	0.055
Dosage of daily magnesium oxide [mg (mean ± SD)]		1,040 ± 430	1,024 ± 453	1,014 ± 509	0.667	0.498
Daily magnesium oxide dosage (≥1,000 mg/<1,000 mg)		145/48	349/124	1,033/466	0.770	0.081
Duration of magnesium oxide treatment [months (mean ± SD)]		31.9 ± 50.4	49.1 ± 55.6	34.1 ± 49.2	0.000**	0.560
Total dosage of magnesium oxide [g (mean ± SD)]		1,019 ± 1,974	1,748 ± 2,621	1,088 ± 1,988	0.000**	0.651
CKD stage	G1 (GFR≥90)	32 (16.6%)	—	239 (15.9%)		
	G2 (90>GFR≥60)	65 (33.7%)	—	696 (46.4%)		
	G3a (60>GFR≥45)	49 (25.4%)	—	379 (25.3%)		
	G3b (45>GFR≥30)	26 (13.4%)	—	148 (9.9%)		
	G4 (30>GFR≥15)	16 (8.3%)	—	34 (8.3%)		
	G5 (14>GFR)	3 (0.2%)	—	5 (2.8%)		
eGFR [ml/min/1.73 m^2^ (mean ± SD)]		63.3 ± 29.8	—	69.9 ± 26.6	—	0.010*
Concomitant use of stimulant laxatives		72	82	257	0.000**	0.000**
Concomitant use of chloride channel activators		11	10	23	0.025*	0.000**

Group A, patients in whom serum concentrations of creatinine or magnesium were not measured; Group B, patients in whom serum concentrations of creatinine, but not magnesium, were measured. ^a^Per Protocol Set vs Group A; ^b^Per Protocol Set vs Group B. CKD was divided into six stages, with G1, G2, G3a, G3b, G4, and G5 defined as ≥90, 60–89, 45–59, 30–44, 15–29, and <15 ml/min/1.73 m^2^, respectively. SD, standard deviation; CKD, chronic kidney disease; eGFR, estimate glomerular filtration rate. **p<0.01, *p<0.05.

**Table 2:** Baseline factors associated with hypermagnesemia

		Hypermagnesemia (Mg >3.0 mg/dl)	Absence of hypermagnesemia (Mg <3.0 mg/dl)	Fisher’s exact test, *p* value	Univariate analysis, OR (95% CI)
Age [older (≥75 years)/younger (<75 years)]		8/2	99/84	0.098^▲^	3.40 (0.70–16.42)
Gender (male/female)		6/4	94/89	0.420	0.70 (0.19–2.58)
Daily magnesium oxide dosage (≥1,000 mg/<1,000 mg)		10/0	135/48	0.053^▲^	N.A.
CKD stage	G1–3b (GFR≥30)/ G4, 5 (30>GFR)	6/4	17/166	0.014*	6.51 (1.67–25.37)**
Concomitant use of stimulant laxatives		6	66	0.119	2.66 (0.72–9.77)
Concomitant use of chloride channel activators		1	10	0.452	1.92 (0.22–16.70)

CKD was divided into six stages, with G1, G2, G3a, G3b, G4, and G5 defined as ≥90, 60–89, 45–59, 30–44, 15–29, and <15 ml/min/1.73 m^2^, respectively. CKD, chronic kidney disease; GFR, glomerular filtration rate; OR, odds ratio; CI, confidence interval. **p<0.01, *p<0.05, ^▲^*p*<0.1.

**Table 3:** Baseline factors associated with high serum magnesium concentration

		High serum Mg concentration (≥2.5 mg/dl)	Normal Mg concentration (<2.5 mg/dl)	Fisher’s exact test, *p* value	Univariate analysis, OR (95% CI)	Multivariate analysis^†^, OR (95% CI)
Age [older (≥75 years)/younger (<75 years)]		22/10	85/76	0.070^▲^	1.97 (0.88–4.42)^▲^	1.93 (0.78–4.78)
Gender (male/female)		14/18	79/82	0.362	0.81 (0.38–1.73)	
Daily magnesium oxide dosage (≥1,000 mg/<1,000 mg)		30/2	115/46	0.004**	6.00 (1.38–26.14)*	12.70 (2.43–66.5)**
CKD stage	G1–3b (GFR≥30)/G4, 5 (30>GFR)	22/10	150/11	0.000***	6.20 (2.36–16.29)***	7.71 (2.46–24.19)***
Concomitant use of stimulant laxatives		17	55	0.035*	2.18 (1.01–4.70)*	1.72 (0.73–4.05)
Concomitant use of chloride channel activators		2	9	0.573	1.13 (0.23–5.47)	

^†^Multivariate logistic regression analysis adjusted for marginally significant factors (*p*<0.1) in univariate analyses. Age, magnesium oxide dosage, CKD G4, 5, and concomitant use of stimulant laxatives were included in the analyses. CKD was divided into six stages, with G1, G2, G3a, G3b, G4, and G5 defined as ≥90, 60–89, 45–59, 30–44, 15–29, and <15 ml/min/1.73 m^2^, respectively. CKD, chronic kidney disease; GFR, glomerular filtration rate; OR, odds ratio; CI, confidence interval. ***p<0.001, **p<0.01, *p<0.05, ^▲^*p*<0.1.
